# Molecular and functional analysis of *phosphoacetylglucosamine mutase* (*PAGM*) and *Glucose-6-phosphate isomerase* (*G6PI*) gene in the white-backed planthopper *Sogatella furcifera* (Hemiptera: Delphacidae)

**DOI:** 10.1038/s41598-026-49624-7

**Published:** 2026-04-20

**Authors:** Zhao Wang, Shao-zhao Qin, Huan Zhu, Gui-Yun Long, Hong Yang, Dao-Chao Jin, Cao Zhou

**Affiliations:** 1 College of Life and Health Science, Kaili University, Kaili, 556011 China; 2 School of Chinese Ethnic Medicine, Guizhou Minzu University, Key Laboratory of Guizhou Ethnic Medicine Resource Development and Utilization in Guizhou Minzu University, State Ethnic Affairs Commission, Guiyang, 550025 China; 3 Institute of Entomology, Guizhou University, Provincial Key Laboratory for Agricultural Pest Management of Mountainous Regions and Scientific Observation and Experimental Station of Crop Pests in Guiyang, Ministry of Agriculture and Rural Affairs of the People’s Republic of China, Guiyang, 550025 China; 4 College of Life Sciences, Chongqing Normal University, Chongqing, 401331 China

**Keywords:** *Sogatella furcifera*, *PAGM*, RNAi, Functional analysis, Biotechnology, Genetics, Molecular biology, Plant sciences

## Abstract

The white-backed planthopper (*Sogatella furcifera*) is a devastating piercing-sucking pest endangering rice production. Genes in the chitin synthesis pathway are critical for its molting and development, representing potential targets for targeted control. This study cloned the full-length cDNAs of *SfPAGM* (KY381946) and *SfG6PI* (KY695112) from *S. furcifera* using RACE-PCR, and systematically explored their molecular characteristics and physiological functions via bioinformatics analysis, qPCR-based spatiotemporal expression detection, and RNAi verification. *SfPAGM* (ORF 1632 bp, encoding 543 amino acids) and *SfG6PI* (ORF 1662 bp, encoding 553 amino acids) shared 88% and 89% identity with homologs in *Nilaparvata lugens*, respectively, showing evolutionary conservation in Delphacidae. Both genes were ubiquitously expressed across the life cycle, with peak expression in newly molted instars and emergent adults, and were most highly expressed in the epidermis but lowest in the head. RNAi results showed that *SfPAGM* dsRNA reduced gene expression by 84% at 72 h, leading to 37.3% mortality in 5 days with molting defects (e.g., old cuticle cracking, wing malformation). *SfG6PI* dsRNA suppressed expression to 58.8% of the control, resulting in 41.3% mortality (5-day survival rate 58.7%) with lethal phenotypes like double-layered cuticle and wing shrinkage. This study confirms the indispensable roles of *SfPAGM* and *SfG6PI* in *S. furcifera*’s molting and growth, providing insights into insect chitin synthesis mechanisms and a theoretical basis for RNAi-based targeted control technologies.

## Introduction

The *Sogatella furcifera* (Horváth), a typical R-strategist rice pest, threatens rice production globally due to its migratory behavior^1,2^. It reduces rice yields directly by feeding on rice nutrients, laying eggs on leaf sheaths, and transmitting multiple rice viruses, including *southern rice black-streaked dwarf virus*, *S. furcifera honeydew virus*, *hepe-like virus*, and *solemo-like virus 1–3*^3–5^. *S. furcifera* undergoes three life stages (egg, nymph, adult) and requires five molts to reach adulthood, during which chitin degradation and synthesis are essential^6,7^, making chitin metabolism pivotal for its growth and survival.

Insect chitin biosynthesis is a complex physiological and biochemical process. Candy and Kilby first proposed this pathway in 1962, starting with glucose and ending with UDP-N-acetylglucosamine (UDP-GlcNAc)^8^; Jaworski later established the conversion from UDP-GlcNAc to chitin using cell-free extracts of *Spodoptera eridania*^9^ and Cohen integrated previous findings to propose the trehalose-to-chitin pathway in 2001^10^. This pathway involves multiple key enzymes, among which phosphoacetylglucosamine mutase (PAGM) and glucose-6-phosphate isomerase (G6PI) are critical but understudied in *S. furcifera*.

PAGM catalyzes the conversion of N-acetylglucosamine-6-phosphate to N-acetylglucosamine-1-phosphate, a key step in UDP-GlcNAc (the direct precursor of chitin) generation^11^. G6PI converts glucose-6-phosphate to fructose-6-phosphate, ensuring the continuity of chitin synthesis. We hypothesize that: (1) SfPAGM regulates chitin biosynthesis by modulating the supply of UDP-GlcNAc, thereby affecting S. furcifera molting, cuticle formation, and survival; (2) SfG6PI maintains the metabolic flux of the chitin synthesis pathway by mediating sugar isomerization, and its dysfunction impairs the growth and development of *S. furcifera*. Despite the reported inhibitory effect of N-acetylglucosamine-6-phosphate on *S. furcifera* growth and the close genetic relationship between *SfG6PI* and *MaG6PI* from Monochamus alternatus^12^, the specific biological functions of *SfPAGM* and *SfG6PI* remain unclear.

Studies in other insects have shown that PAGM expression is regulated by genes in the chitin synthesis pathway^13–16^, highlighting its core role. Research on *SfPAGM* and *SfG6PI* is of great significance for developing RNAi-based green pesticides: as key enzymes in chitin synthesis (a process unique to insects and absent in plants and mammals), they are ideal targets for pest control. Silencing these genes via RNAi can specifically inhibit *S. furcifera* growth and survival without polluting the environment or harming non-target organisms, addressing the drawbacks of traditional chemical pesticides.

Therefore, this study aims to characterize *SfPAGM* and *SfG6PI* by cloning their full-length cDNAs, analyzing their molecular features and expression patterns, and verifying their biological functions using RNAi. The findings will fill the research gap in chitin synthesis-related enzymes in *S. furcifera* and provide a theoretical basis for developing efficient, green RNAi-based pesticides against this pest.

## Results

### Identification and characterization of SfPAGM and SfG6PI

**Fig. 1:**
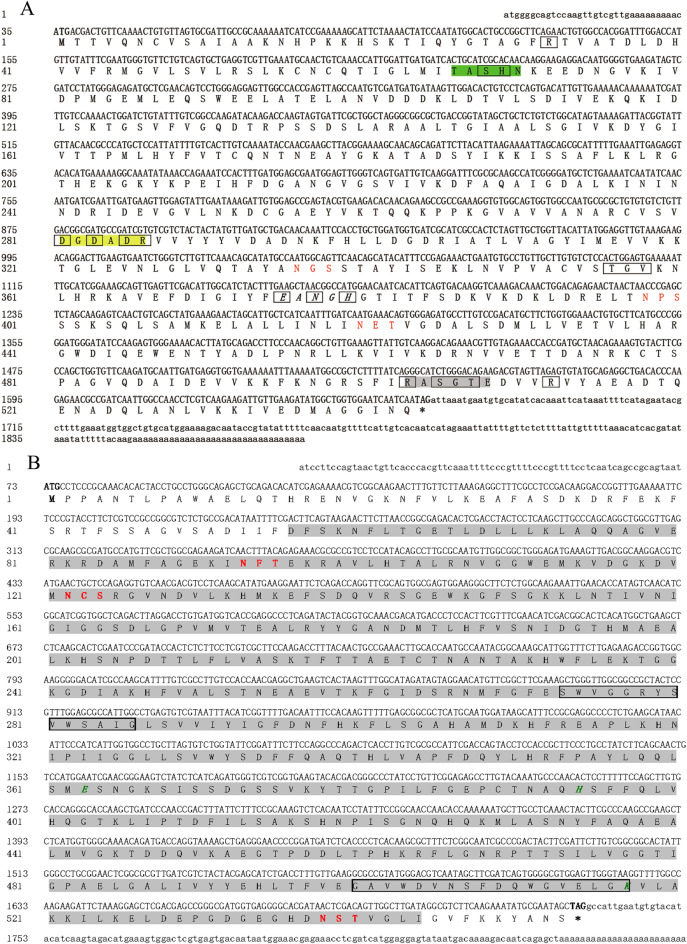
Nucleotide sequence, deduced amino acid sequence, and three-dimensional structural model of *SfPAGM* and *SfG6PI* from *S. furcifera*. *Note*: A: Nucleotide sequence, deduced amino acid sequence of *SfPAGM*；B: Nucleotide sequence, deduced amino acid sequence of *SfG6PI*; The initiation codon (ATG) is shown in boldface, while the termination codon (TAG) is marked in bold with an asterisk.

The full-length cDNAs of *SfPAGM* and *SfG6PI* were both isolated from *S. furcifera* using RACE-PCR techniques, with their GenBank accession numbers assigned as KY381946 and KY695112, respectively. A comparative analysis of their cDNA sequences revealed subtle differences in length and structural composition: the *SfPAGM* cDNA is 1882 bp in length (Fig. 1A), while the *SfG6PI* cDNA is slightly shorter at 1873 bp (Fig. 1B). Both cDNAs consist of a 5′ untranslated region (UTR), a 3′ UTR, and a complete open reading frame (ORF), but their UTR lengths and ORF sizes differ significantly. Specifically, the *SfPAGM* cDNA contains a 1632 bp ORF, flanked by a 34 bp 5′ UTR and a 216 bp 3′ UTR. In contrast, the *SfG6PI* cDNA harbors a longer ORF of 1662 bp (encoding 553 amino acid residues), accompanied by a 72 bp 5′ UTR and a shorter 3′ UTR of 139 bp.

The putative proteins encoded by SfPAGM and SfG6PI also exhibit distinct physicochemical properties. The SfPAGM protein has a predicted molecular mass of 59.17 kDa and an isoelectric point (pI) of 5.67, whereas the SfG6PI protein shows a slightly higher molecular weight of 60.99 kDa and a more neutral pI of 6.50.

Bioinformatic analyses using the NetNGlyc 1.0 Server identified three conserved N-glycosylation motifs in both SfPAGM and SfG6PI proteins; for SfG6PI, these sites were specifically mapped to N93, N122, and N538 (Fig. 1A). Additionally, ScanProsite analysis revealed two conserved amino acid motifs (SWVGGRYSVWSAIG and GAVWDVNSFDQWGVELGK) in SfG6PI (Fig. 1B). Notably, neither SfPAGM nor SfG6PI contained a signal peptide or transmembrane helix domain, as confirmed by transmembrane topology prediction and signal peptide scanning, suggesting that both proteins are likely soluble and function in the cytoplasm rather than being membrane-bound or secreted.

### Sequence comparisons and phylogenetic analysis

**Fig. 2:**
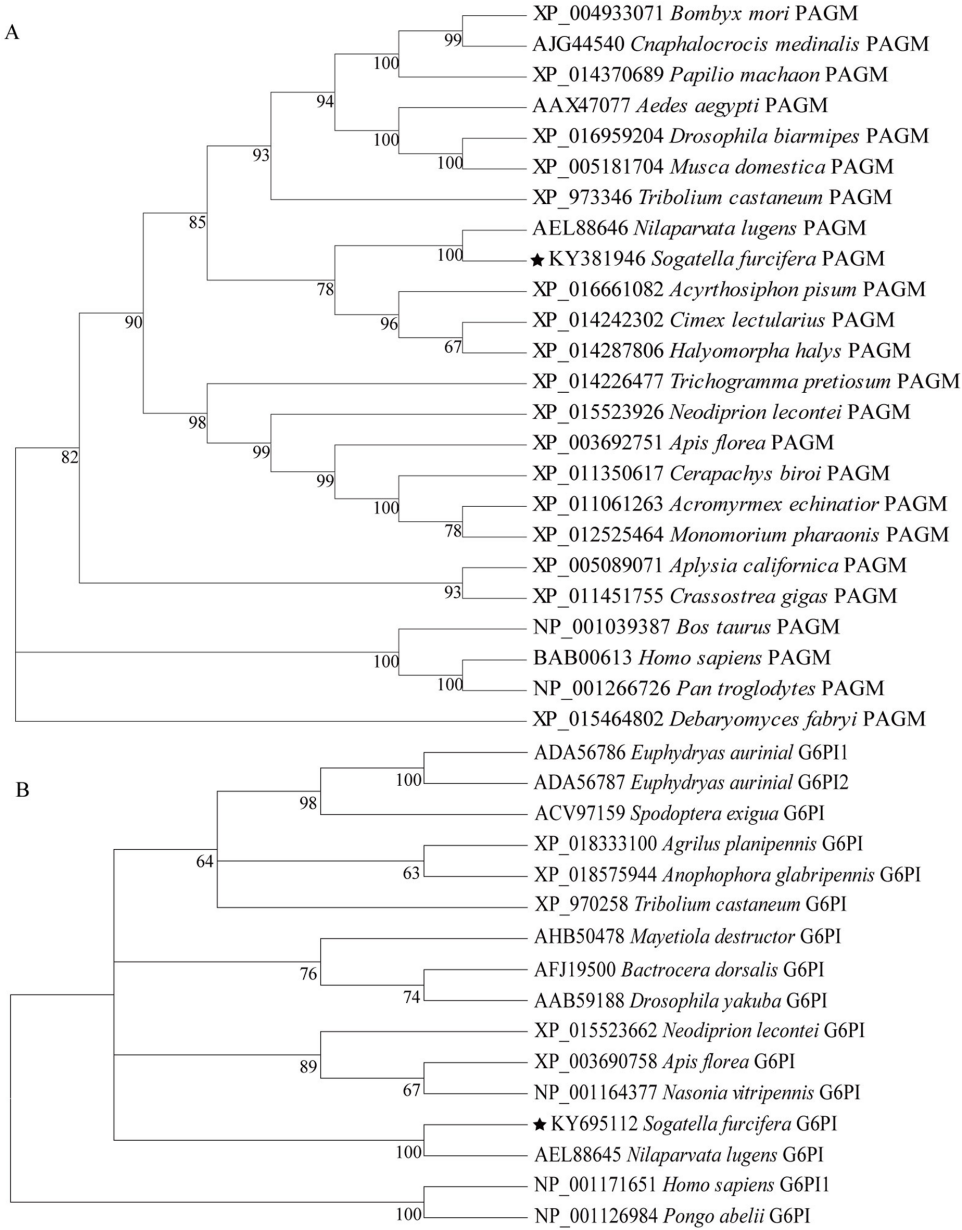
Phylogenetic tree. *Note*: A: Phylogenetic tree of SfPAGM; B: Phylogenetic tree of SfG6PI; The tree was constructed using MEGA 6 via the neighbor-joining method. Bootstrap analyses of 1000 replicates were performed and bootstrap values are shown next to the branches.

BLAST alignment showed that the amino acid sequence of *SfPAGM* shared 88%, 59%, 58%, and 56% identity with those of *N. lugens* (AEL88646), *Zootermopsis nevadensis* (KDR17892), *Tribolium castaneum* (XP_973346), and *Aedes aegypti* (AAX47077), respectively (Fig. 2A). *SfG6PI* had the highest similarity (89%) with *N. lugens* G6PI (AEL88644)(Fig. 2B). Phylogenetic analysis (MEGA 6.06, neighbor-joining method) indicated that *SfPAGM* and *SfG6PI* each clustered closely with their respective homologs from *N. lugens* (Fig. 2A and B), consistent with their traditional taxonomic classification.

### Spatio-temporal expression profile of SfPAGM and SfG6PI

**Fig. 3:**
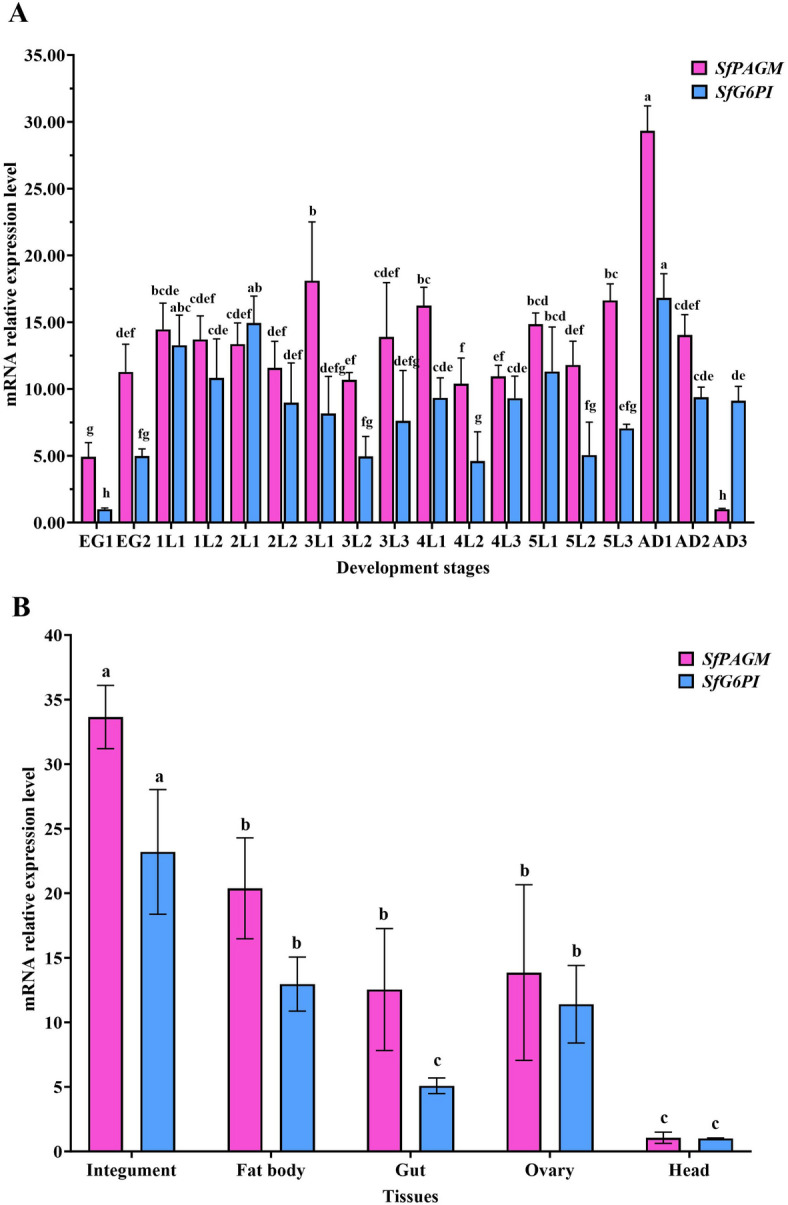
Relative expression levels. *Note*: A:Expression levels of *SfPAGM* and *SfG6PI* in *S. furcifera* at different developmental stages; B: Expression levels of *SfPAGM* and *SfG6PI* in different tissues of *S. furcifera*; Relative expression values were determined by setting the lowest expression level to 1 as a reference. All measurements are based on three biological replicates, with data presented as mean ± SE. Developmental time points are abbreviated: EG1 (first day of eggs), lL1 (first day of first-instar nymphs), AD1 (first day of adults). Statistical significance (*P* < 0.05) was assessed via one-way ANOVA with Duncan’s multiple range test, with differing lowercase letters above bars indicating significant differences.

Following RNA extraction from samples collected at 18 developmental time points (egg to adult), qPCR was used to quantify *SfPAGM* and *SfG6PI* expression (Fig. 3). Data confirmed that these two genes are expressed at all stages of the life cycle. Specifically, the expression levels of both *SfPAGM* and *SfG6PI* were higher on the first day of each age stage, except for the eggs. The expression of *SfPAGM* and *SfG6PI* reached a peak on the initial day of newly emerged adulthood and notably declined in the subsequent stages of adult life (Fig. 3A ).

Notably, qPCR analysis of five *S. furcifera* tissues (integument, fat body, intestine, ovary, head) revealed striking tissue-specific differences in *SfPAGM* and *SfG6PI* expression. The integument displayed the highest expression, the head the lowest, while fat body, intestine, and ovary showed statistically indistinguishable levels (Fig. 3B).

### Functional analysis of SfPAGM and SfG6PI

**Fig. 4:**
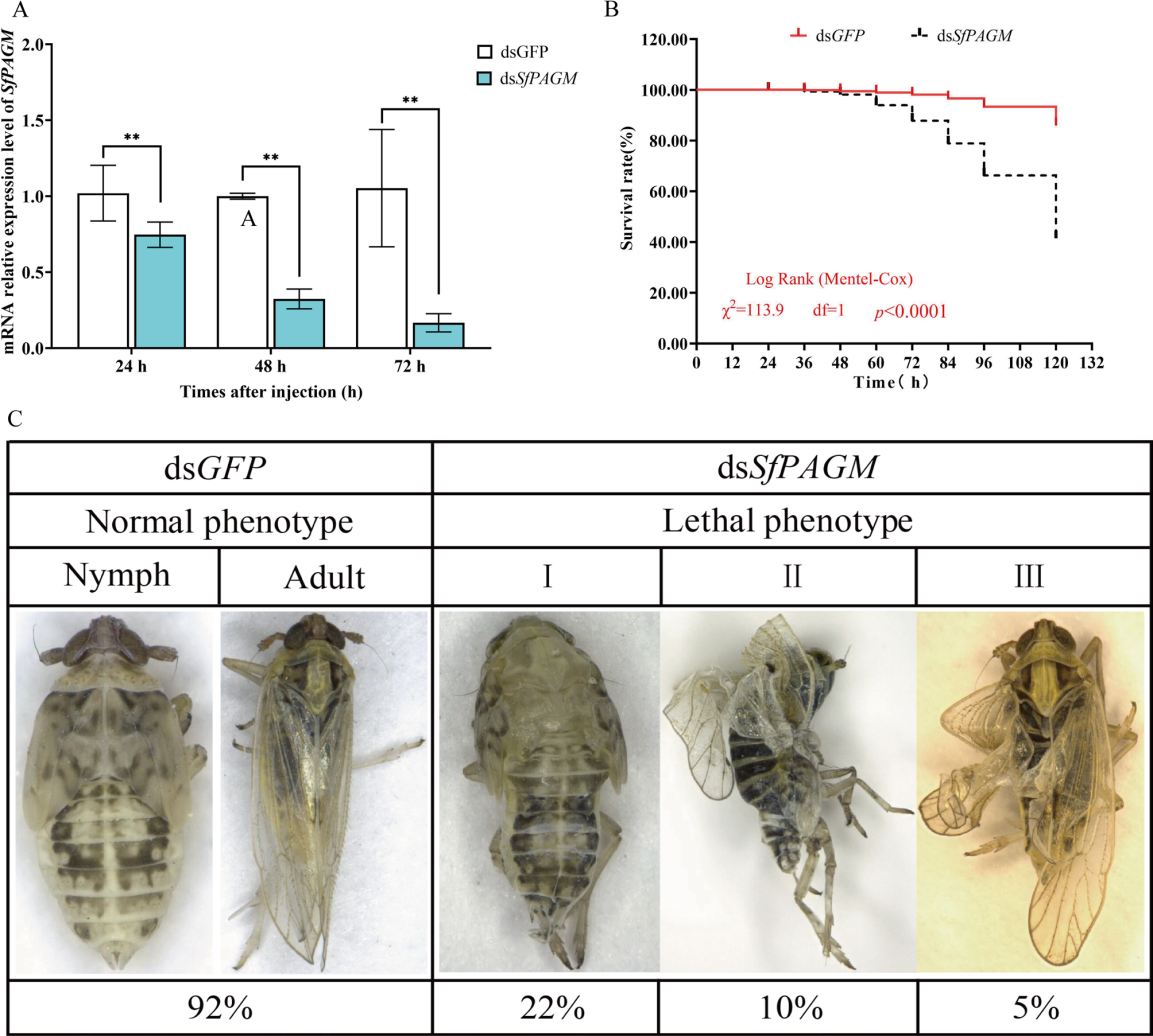
Target gene expression level, mortality and phenotype of *S. furcifera* subjected to *SfPAGM* dsRNA injection. *Note*: A: RNAi interference efficiency of *SfPAGM*; B: Survival rate of *S. furcifera* after *SfPAGM* RNAi; C:  Phenotype of *S. furcifera* after *SfPAGM* RNAi. Data are presented as the mean ± standard error (SE) from three biological replicates. Denotes statistically significant differences at *P* < 0.01 (Independent Samples t-test). This panel illustrates the survival rate of insects following dsRNA injection into 1-day-old fifth-instar nymphs, with each nymph receiving 100 ng of dsRNA. Data represent the mean ± SE from three biological replicates, with fifty insects per experimental group.

To better understand *SfPAGM*’s physiological role, specific dsRNA was synthesized in vitro and injected into 1-day-old fifth-instar nymphs. Strikingly, the transcription level of *SfPAGM* gradually decreased after injection with *SfPAGM* dsRNA, and was down-regulated by 84% relative to the control group at 72 h (Fig. 4A) (t = 3.932, df = 4, *p* = 0.0085).

After dsRNA injection, the survival of the test insects was observed continuously, with the results presented in Fig. 4B. The survival pattern was found to be highly similar to that following *SfPAGM*-dsRNA injection: the survival rate of insects in the *SfPAGM*-dsRNA group began to decrease markedly from the second day, reaching 74% on the third day. With the prolongation of time, the survival rate continued to decline, falling to 62.7% on the fifth day.

After successful knockdown of the target gene, three aberrant phenotypes were observed in both nymphal and adult stages (Fig. 4C). The first phenotype was characterized by a distinct split in the old cuticle covering the head and thorax, with the remainder of the body retained within the exoskeleton, culminating in mortality (Fig. 4C-I). Second, incomplete detachment of the old cuticle from the body was observed, leading to wing malformations; additionally, adults in the injected group exhibited a significantly reduced body size compared to normal individuals (Fig. 4C-II). Lastly, the injected nymphs were able to metamorphose into adults, but their wings failed to properly unfold (Fig. 4C-III). The findings suggest that *SfPAGM* is indispensable for proper growth and molting in *S. furcifera*, given that its suppression results in severe morphological abnormalities and death.

**Fig. 5:**
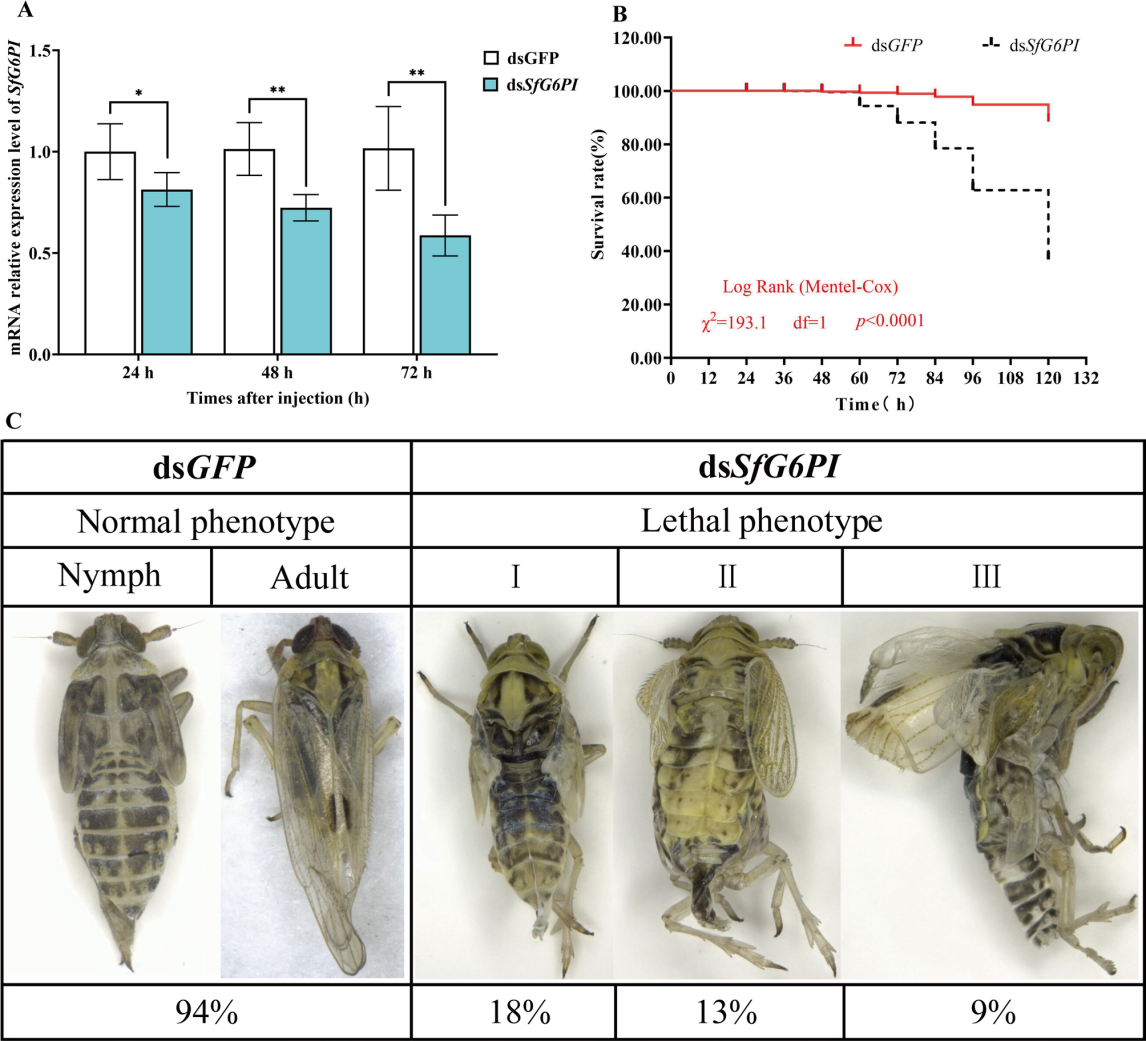
Target gene expression level, mortality and phenotype of *S. furcifera* subjected to *SfG6PI* dsRNA injection. *Note*: A: RNAi interference efficiency of *SfG6PI*; B: Survival rate of *S. furcifera* after *SfG6PI* RNAi; C:  Phenotype of *S. furcifera* after *SfG6PI* RNAi.Data are presented as the mean ± standard error (SE) from three biological replicates. Denotes statistically significant differences at *P* < 0.01 (Independent Samples t-test). This panel illustrates the survival rate of insects following dsRNA injection into 1-day-old fifth-instar nymphs, with each nymph receiving 100 ng of dsRNA. Data represent the mean ± SE from three biological replicates, with fifty insects per experimental group.

dsRNA targeting *SfG6PI* was injected into the 1st - day 5th instar nymphs of *S. furcifera*. It was found that the expression level of the target gene in the *SfG6PI* - dsRNA injection group was significantly down-regulated (*P* < 0.05) (Fig. 5A), accounting for 58.8% of that in the ds*GFP* injection group. This indicated that during the chitin synthesis process in insects, the transcriptional level might decrease due to the silencing of the *SfG6PI* gene, thereby affecting the normal synthesis of chitin.

After dsRNA interference treatment, the survival of *S. furcifera* nymphs was affected to a certain extent (Fig. 5B). Specifically, when dsRNA was injected into first-day 5th-instar nymphs, the survival rate of insects in the *SfG6PI*-dsRNA injection group exhibited a significant downward trend starting from the second day, reaching 73.3% on the third day. As time progressed, the survival rate continued to decline, falling to 58.7% on the fifth day.

Observation of the developmental status of nymphs after dsRNA injection into 5th instar individuals revealed that the *SfG6PI*-dsRNA injection group exhibited two major types of phenotypic defects: molting defects and molting-wing expansion defects (Fig. 5C)). Specifically: ① Molting defects (e.g., Phenotype I): 18% of the test insects showed complete separation of the old and new cuticles, with the old cuticle on the head and thorax already split, but the insect body failed to shed the old cuticle normally, resulting in a double-layered cuticle structure. All insects with this defect eventually died. ② Molting-wing expansion defects (e.g., Phenotypes II and III): the insect body had partially detached from the old cuticle, but the cuticular appendages on the abdomen and appendages could not be completely shed; meanwhile, the formed wings failed to extend normally and appeared shriveled. All insects with this defect eventually died.

## Discussion

Chitin synthesis is an essential metabolic process for insect growth and development, as chitin constitutes the primary structural component of the exoskeleton, peritrophic matrix, and tracheal system. In this study, we cloned and functionally characterized the *SfPAGM* and *SfG6PI* of *S. furcifera*, integrating sequence analysis, spatiotemporal expression profiling, and RNA interference (RNAi)-mediated functional validation to clarify their roles in the chitin synthesis pathway and provide insights into their potential as pest control targets.

Sequence analysis revealed distinct molecular characteristics of *SfPAGM* and *SfG6PI*, which are consistent with their evolutionary conservation and functional specialization. *SfPAGM* encodes a 543-amino acid protein, with an amino acid length similar to its homologs in *(A) aegypti* (*AePAGM*, 549 amino acids) and *(B) mori* (*BmPAGM*, 548 amino acids)^12,17^, while showing significant interspecific variation compared to *L. migratoria LmPAGM* (497 amino acids)^18^. Phylogenetic and sequence similarity analyses demonstrated that SfPAGM shares the highest amino acid identity (96.98%) with its ortholog in *N. lugens*, whereas its similarity with *Ae. aegypti* is only 55.51%—a pattern consistent with the 56% identity between *LmPAGM* and *Ae. aegypti*^18^. This divergence indicates that PAGM has undergone adaptive evolution among different insect taxa, particularly within planthoppers. Bioinformatics analysis further showed that *SfPAGM* possesses a tertiary structure consisting of 18 α-helices, 19 β-sheets, and 4 functional domains, with four specific loops adjacent to the active center (serine loop and metal-binding loop) speculated to regulate substrate orientation and catalytic efficiency—features highly consistent with the typical catalytic domain of phosphatase proteins (predicted by SWISS-MODEL). Additionally, *SfPAGM* contains a conserved phosphoglucomutase 3 (PGM3) domain (residues 25–531), which is also present in *BmPAGM* (residues 13–544) 20, confirming functional conservation of PAGM across insect species.

In contrast, *SfG6PI* exhibits high structural stability (instability index = 19.89 < 40) and hydrophilicity (GRAVY = − 0.246), with high glycine (9.2%) and leucine (8.5%) contents that may contribute to protein stability. Three potential N-glycosylation sites (N93, N122, N538) suggest its involvement in signal transduction or substrate binding through post-translational modifications. Phylogenetically, *SfG6PI* shares an even higher homology (89%) with its *N. lugens* ortholog than *SfPAGM* (88%), and maintains high homology across diverse insect taxa—indicating that as a key enzyme in glucose metabolism, *SfG6PI* is subject to stronger functional constraints than *SfPAGM*. The clustering of both *SfPAGM* and *SfG6PI* with *N. lugens* homologs in phylogenetic trees is consistent with the traditional taxonomic classification of Delphacidae in Hemiptera, highlighting evolutionary conservation of these two genes among planthoppers.

The spatiotemporal expression patterns of *SfPAGM* and *SfG6PI* further reflect their core roles in chitin synthesis and insect molting. Both genes are expressed throughout the entire life cycle of *S. furcifera*, with expression peaks on the first day of each instar (except the egg stage) and in newly emerged adults—an expression profile highly synchronized with the critical period of chitin synthesis during the molting cycle, when new cuticle formation requires abundant chitin precursors and synthesis-related enzymes. Tissue-specific expression analysis showed that both genes are most highly expressed in the cuticle and least expressed in the head, which directly correlates with their function in cuticular chitin synthesis: the insect cuticle, composed primarily of chitin and proteins, serves as a physical barrier, and chitin synthesis efficiency directly impacts cuticle formation and molting success. Specifically, *SfG6PI*, as a glucose-6-phosphate isomerase, likely regulates the glucose metabolism pathway to supply N-acetylglucosamine (the basic structural unit of chitin) precursors, while the phosphatase activity of *SfPAGM* may be involved in phosphate group transfer or signal regulation during chitin synthesis. These expression patterns are consistent with previous reports in *B. mori* and *L. migratoria*, where PAGM exhibits periodic expression peaks during premolting and molting stages, followed by a decrease during intermolt stages^17,18^. Notably, *SfPAGM* expression in the epidermis was 31.7-fold higher than in the head, further confirming its essential role in chitin synthesis and molting regulation in *S. furcifera*.

RNAi-mediated functional validation confirmed the irreplaceable roles of *SfPAGM* and *SfG6PI* in the growth and development of *S. furcifera*. Injection of *SfPAGM*-specific dsRNA into 1-day-old fifth-instar nymphs resulted in an 84% silencing efficiency, leading to a 37% mortality rate within 5 days and phenotypic defects including incomplete ecdysis (failed separation of the old cuticle), body deformation, wing malformations, and reduced body size—phenotypes characteristic of chitin synthesis deficiency. These findings align with previous studies showing that knockdown of chitin biosynthesis genes (Tre, UAP, GFAT) in *S. furcifera* induces similar molting defects and adult deformities^19–21^, and are consistent with cross-species observations in *L. migratoria*, where silencing *LmPAGM* resulted in a 30% adult emergence failure rate^18^. For *SfG6PI*, RNAi treatment achieved a 41.2% silencing efficiency, reducing the mortality of 41.3% within 5 days and causing defects such as double-layered cuticle and wing shrinkage—further verifying its core role in supplying chitin precursors: gene silencing leads to insufficient raw materials for chitin synthesis, incomplete new cuticle formation, and eventual molting failure. These results differ from studies on *B. dorsalis*, where knockdown of *BdG6PI* did not affect larval-pupal molting^22^, suggesting species-specific functional differences in *G6PI* among insect orders. Chen et al.^23^ reported that *BdG6PI* may be involved in midgut chitin synthesis in *B. dorsalis*, as its expression and midgut chitin content were significantly downregulated under starvation conditions—indicating that *G6PI*’s role in chitin synthesis may vary by tissue and insect species.

The low interference efficiency of *SfG6PI* may be attributed to off-target effects. RNA interference (RNAi) can specifically suppress the expression of target genes in pests. However, double-stranded RNA (dsRNA) carries potential off-target effects. dsRNA can induce off-target silencing through high sequence similarity with non-target genes. For example, the risk of off-target effects is low when sequence identity is below 67%; off-targeting is possible within the range of 67–78%; and the probability of off-targeting increases significantly when it is above 78%^28^. In the present study, we only reduced the likelihood of off-target effects through sequence alignment. Therefore, it is recommended that off-target effects be fully considered in subsequent RNAi experiments. Multiple pairs of RNAi primers should be designed in different regions to synthesize dsRNA for interference, or the concentration of dsRNA should be increased, since high-concentration dsRNA can reduce the risk of off-target effects. Studies have shown that protecting dsCYP6BQ6 with Lipofectamine 2000 does not significantly enhance the silencing effect on other genes, indicating that the injection dose is sufficient and that dsRNA degradation has little effect on off-target silencing^24^.

Notably, in the present study, the 72 h interference efficiency of *SfG6PI* (41.2%) was much lower than that of *SfPAGM* (84%), whereas its lethal mortality was 4% points higher than that of *SfPAGM* RNAi. This difference is essentially attributed to the distinct metabolic positions, functional essentiality and redundancy of the two genes, which are further verified by studies on insect energy metabolism and chitin synthesis pathways. G6PI, as a key bifunctional enzyme, is located at the core of both glycolysis and chitin synthesis pathways, and its function is irreplaceable in insect growth and development^25^. It catalyzes the reversible isomerization between glucose-6-phosphate and fructose-6-phosphate, which is not only a prerequisite for glycolysis to produce adenosine triphosphate (ATP)—the direct energy source for all cellular activities—but also provides essential precursors for chitin synthesis in insects^26^. For example, in *B. dorsalis*, *G6PI* is widely expressed in various tissues and stages, and its normal activity is crucial for maintaining glucose homeostasis and energy supply. When *G6PI* gene expression is interfered with by RNAi, glycolysis is completely blocked, resulting in rapid depletion of intracellular ATP reserves. This directly leads to the stagnation of vital physiological processes such as protein synthesis, cell division, ion transport and nerve conduction, and ultimately causes acute death of insects within a short period.

In sharp contrast, PAGM only participates in the specific biosynthetic pathway of chitin, and its functional influence is relatively limited with potential compensatory mechanisms. PAGM acts in the hexosamine biosynthetic pathway (HBP), catalyzing the conversion of N-acetylglucosamine-6-phosphate to N-acetylglucosamine-1-phosphate, which serves as a precursor for UDP-N-acetylglucosamine synthesis and further supports chitin formation in insect cuticle, peritrophic membrane and tracheal system^27^. However, unlike *G6PI*, *PAGM* is not a rate-limiting enzyme in the chitin synthesis pathway, and the HBP pathway can be partially regulated by other enzymes such as glutamine: fructose-6-phosphate amidotransferase (GFAT)^25^. In addition, some insects can alleviate the deficiency caused by *PAGM* silencing by ingesting small amounts of amino sugars from their diet. More importantly, chitin synthesis in insects is mainly active during molting cycles rather than being continuously essential, so the lethal effect of *PAGM* RNAi usually appears in the molting stage as subacute death symptoms such as molting difficulty and cuticle fragility, resulting in a lower overall mortality rate compared with *G6PI* interference.

The difference in functional redundancy between *G6PI* and *PAGM* further exacerbates the mortality gap after RNAi. *G6PI* is generally encoded by a single-copy gene in most insects, with high sequence conservation and no functional analogs that can replace its role in energy metabolism. This means that even a partial reduction in *G6PI* expression can lead to severe metabolic disorders and high mortality. On the contrary, *PAGM* may have multiple family members or functional homologs in some insect species. For instance, in *T. castaneum*, studies on chitin synthesis-related genes have shown that silencing a single *PAGM* subtype may not completely block chitin synthesis due to functional compensation from other homologous genes^28^. Even in the absence of homologous gene compensation, the lethal priority of chitin synthesis defects is much lower than that of energy metabolism interruption, so the mortality rate of insects after PAGM interference is significantly lower.

Relevant RNAi studies on insect pest control have also confirmed this phenomenon. For example, in the RNAi experiment on *L. migratoria*, silencing the *G6PI* gene resulted in a mortality rate of more than 80% within 48 h, while silencing the *PAGM* gene only caused a mortality rate of less than 50% even after 72 h, with most deaths occurring during molting^26^. Similar results were observed in *C. medinalis*, where interference with genes related to energy metabolism caused more rapid and severe lethal effects than interference with chitin synthesis pathway genes^15^. These findings collectively indicate that the core reason for the higher mortality rate after *G6PI* interference is its irreplaceable role in energy metabolism, while *PAGM*, as a gene involved in specific biosynthetic pathways, has limited influence and potential compensatory mechanisms.

Collectively, our results fill the gap in research on the chitin synthesis mechanism of *S. furcifera*, as this is the first study to clarify the functions of S*fPAGM* and *SfG6PI* in this species. Both genes possess core characteristics of ideal green pesticide targets: the chitin synthesis pathway is unique to insects (absent in mammals and plants), ensuring high species specificity and avoiding impacts on non-target organisms; additionally, the RNAi technology offers high efficiency and environmental friendliness, making it a promising bioengineering approach for pest control. The significant phenotypic defects induced by silencing *SfPAGM* and *SfG6PI* provide direct evidence for the development of targeted RNAi pesticides against *S. furcifera*. Compared with existing studies, silencing of orthologous genes in *N. lugens* also caused similar molting defects, suggesting that these two genes could serve as shared targets for the synergistic control of planthopper pests.

In conclusion, our sequence analysis, expression profiling, and RNAi results demonstrate that *SfPAGM* and *SfG6PI* synergistically regulate chitin synthesis and molting in *S. furcifera*, with *SfPAGM* playing a central role in catalytic reactions of chitin precursors and *SfG6PI* contributing to precursor supply through glucose metabolism. These findings not only enhance our understanding of the molecular mechanisms underlying chitin synthesis in planthoppers but also provide new candidate targets for the development of environmentally friendly pest control strategies. Future studies should focus on exploring the regulatory networks of these two genes in the chitin synthesis pathway and optimizing RNAi delivery systems to improve silencing efficiency and pest control efficacy.

Although phenotypic observations have confirmed the essential roles of *SfPAGM* and *SfG6PI* in chitin biosynthesis in *S. furcifera*, the present study lacks determinations of corresponding enzyme activities and detailed examinations of cuticular structural alterations following RNAi-mediated silencing of these two genes. Future investigations should focus on evaluating the impacts of target gene silencing on downstream enzyme activities. Moreover, paraffin sectioning and scanning electron microscopy are recommended to characterize the cuticular ultrastructure, which will contribute to a more comprehensive understanding of the biological functions of *SfPAGM* and *SfG6PI*.

## Conclusion

This study identified two key chitin synthesis-related genes, *SfPAGM* and *SfG6PI*, in *S. furcifera*, and clarified their molecular characteristics, evolutionary conservation, spatiotemporal expression patterns, and physiological functions. RNAi experiments confirmed that both genes are crucial for insect molting and growth, and their silencing can induce lethal phenotypes. These findings not only enrich the research on the molecular mechanisms of the insect chitin synthesis pathway but also provide candidate targets and scientific basis for the development of targeted and environmentally friendly planthopper control technologies.

## Materials and methods

### Insect rearing

White-backed planthoppers were sourced from Guiyang’s rice fields in Guizhou Province (2013) and continuously propagated on TN1 rice seedlings within a controlled-environment growth chamber for successive generations. The rearing environment was set to a 16-hour light/8-hour dark cycle, with temperature maintained at 25 ± 1℃ and humidity at 75%±5^29,30^.

### Total RNA isolation and cDNA synthesis

Following the protocol provided with TRIzol reagent (Invitrogen, Carlsbad, California, USA), total RNA was extracted. RNA integrity was confirmed through 1% agarose gel electrophoresis, while a Nanodrop 2000 spectrophotometer (Thermo Fisher Scientific, Wilmington, Delaware, USA) was used to assess RNA concentration and purity. Thereafter, oligo-dT primers and the AMV First Strand cDNA Synthesis Kit (Sangon Biotech, Shanghai, China) were employed to synthesize first-strand cDNA.

### Primer design

First, partial *SfPAGM* and *SfG6PI* nucleotide sequences were retrieved from the *S. furcifera* transcriptome (SRP116252). Next, Primer Premier 6.0 software was used to design the primers (Table 1). The *SfRPL9* (*ribosomal protein L9*) and *SfTUB* (*alpha 1-tubulin*) genes of *S. furcifera* served as internal reference genes^30^. The synthesis of all primers was commissioned to Sangon Biotech (Shanghai) Co., Ltd. (Shanghai, China).

**Table 1:** Primers used in the study.

Gene name and GenBank accession number	cDNA Fragment	Primer name	Primer sequence (5′-3′)
*SfPAGM*	PCR1	PAGM-F1	ACAAGGAAGAGGACAATGG
KY381946	5′RACE	PAGM-R1	CAGCATCAACATAGTAGTAGAC
		PAGM-51	CAGCGAATCACTACTTGGTCTTG
	3′RACE	PAGM-52	CCATTGTCCTCTTCCTTGTTGTG
		PAGM-31	GAAACCACCGATGCTAAC
	ORF confirmation	PAGM-32	CGTAGTTAGAGTGTATGC
		PAGM-F	CATGACGACTGTTCAAAACTGT
		PAGM-R	CTATTGATTGATTCCACCAGCC
*SfG6PI*	PCR1	SfG6PI-F1	GGTGGCTCAGACTTAGGA
		SfG6PI-R1	TACAAGGCTCTCCGAACA
	5′RACE	5’G6PI-R1	TAAGTCTGAGCCACCGAT
		5’G6PI-R2	CAAGGCTGTATGGAGGAC
	3′RACE	3’G6PI-F1	TGTTCGGAGAGCCTTGTA
		3’G6PI-F2	TACTACGAGCATCTGACCT
	ORF confirmation	SfG6PI-F	TATGCCTCCCGCAAACACA
		SfG6PI-R	CAATGGCCTAGCTATTCGC
*PRL9* KM885285.1 *TUB* KP735521.1	qPCR synthesis	qPAGM-F	GTCGTCTACTACTATGTTG
		qPAGM-R	CGGAAATGTATGCTGTTG
		qG6PI-F	GCTGGGAGATGAAAGTTGA
		qG6PI-R	ATGCCGATGTTGACTATGG
		q*PRL9-*F	GTGAACAAGTGCGAAGGA
		q*RPL9-*R	TCATAGCAGTGCGTCAAC
		q*TUB-*F	CGCTGTTGATGGAGAGGCTGTC
		q*TUB-*R	ACGACGGCTGTGGATACCTGTG
	dsRNA synthesis	dsPAGM-F	*TAATACGACTCACTATAGGG*CGTGTCGTCTACTACTATGT
		dsPAGM-R	*TAATACGACTCACTATAGGG*TCTGTTAGCATCGGTGGTT
		dsG6PI-F	*TAATACGACTCACTATAGGG*CTTAGTGTCTGGTATTCGGATT
		dsG6PI-R	*TAATACGACTCACTATAGGG*AAGGTCAGATGCTCGTAGTA
GFP		dsGFP-F	*TAATACGACTCACTATAGGG*AAGGGCGAGGAGCTGTTCACCG
U73901.1		dsGFP-R	*TAATACGACTCACTATAGGG*CAGCAGGACCATGTGATCGCGC

Underlined nucleotides indicate DNA sequences transcribed downstream of the T7 promoter. qPCR, quantitative real-time polymerase chain reaction; dsRNA, double-stranded RNA.

### Cloning of SfPAGM and SfG6PI cDNAs

Amplification of the full-length *SfPAGM* and *SfG6PI* cDNA was carried out with the primer pairs in Table 1. Using first-strand cDNA as template, PCR was run, and amplicons were separated via agarose gel electrophoresis. Subsequent steps involved excising target bands, purifying them, ligating into the pMD18-T vector, and transforming into competent *E. coli* DH5α cells. The 25-µL PCR mixture consisted of 2.0 µL cDNA template, 1.0 µL of each 10 µM primer, 2.5 µL 10× LA PCR Buffer (with Mg²⁺), 4.0 µL dNTP Mix (2.5 mM per nucleotide), 0.25 µL LA Taq DNA Polymerase, and 14.25 µL nuclease-free water.

PCR thermal cycling began with denaturation at 94 °C for 3 min, followed by 30 cycles consisting of 94 °C for 30 s (denaturation), 55 °C for 30 s (annealing), and 72 °C for 1 min (extension), and concluded with a 10-min extension at 72 °C. Amplified products were separated by gel electrophoresis, after which the target band was gel-purified, ligated into a cloning vector, and introduced into competent cells. Positive colonies were validated via Sanger sequencing performed by Sangon Biotech (Shanghai, China).

### Sequence analysis

The full-length of the Sequenced fragments were assembled using SeqMan software to obtain the full-length cDNAs of *SfPAGM* and *SfG6PI*. Nucleotide sequences were edited with DNAMAN 7.0 (Lynnon Biosoft, California, USA). To explore sequence similarities and homologies, we utilized the NCBI BLAST program (https://blast.ncbi.nlm.nih.gov/Blast.cgi). Subsequent protein analyses involved specialized tools: NCBI ORF Finder for open reading frame identification, ExPASy ProtParam for physicochemical property calculation, NetNGlyc 1.0 for N-glycosylation site prediction, TMHMM v.2.0 for transmembrane helix detection, and SignalP 4.1 for signal peptide prediction. The 3D structure of SfPAGM was predicted using SWISS-MODEL (https://www.swissmodel.expasy.org/interactive) and visualized with PyMOL 1.1. Phylogenetic relationships were inferred in MEGA 6.06 via the neighbor-joining method, with 1000 bootstrap replicates to test branch support.

### Developmental and tissue expression analysis of SfPAGM and SfG6PI

To characterize the developmental expression profile of *SfPAGM* and *SfG6PI*, samples representing the entire life cycle—including eggs, nymphs, and adults—were collected. In the tissue-specific expression analysis, five types of tissues—integument, fat body, gut, ovary, and head—were micro-dissected from 1-day fifth-instar nymphs and 3-day adults, with each tissue having three biological replicates. Total RNA from the tissues or whole bodies was isolated using Omega bio-tek’s HP Total RNA Kit (Norcross, GA, USA), which includes gDNA removal columns. First-strand cDNA was synthesized using the AMV RT reagent Kit (Sangon Biotech) with oligo-dT primers. Gene-specific primers for qPCR are detailed in Table 1.

Before conducting formal qPCR tests, a combination of RNA samples from various developmental phases and tissues was utilized to generate cDNA for validating primer efficiency, with the aim of ensuring that amplification efficiencies remained within the 90–110% range. Real-time quantitative PCR was carried out on a Bio-Rad CFX-96 system (manufactured by Bio-Rad in Hercules, CA, USA). Each 20-µL reaction mixture consisted of 10 µL of FastStart Essential DNA Green Master (supplied by Roche Diagnostics in Shanghai, China), 1 µL of cDNA template, 1 µL (10 µM) of each primer, and 7 µL of RNase-free water. The thermal cycling protocol included an initial denaturation step at 95 °C for 10 min, followed by 40 cycles of denaturation at 95 °C for 30 s and annealing at 55 °C for 30 s. After the amplification process, a melting curve analysis (over a temperature range of 65–95 °C) was performed to confirm the specificity of the amplicons. The relative expression levels of the genes were determined using the 2^−ΔΔCt^ method, as described by Livak and Schmittgen^31^.

### Double-stranded RNA preparation

Off-target effects are a common issue in RNAi research. Sequence alignment of *SfPAGM* and *SfG6PI* was performed using the BLASTp program. RNAi primers encompassing typical functional domains were designed in regions with a sequence identity threshold of ≥ 85% to minimize off-target effects. Based on the previously acquired *SfPAGM* and *SfG6PI* sequence (Table 1), gene-specific primers were engineered. These primers had the T7 polymerase promoter sequence integrated at their 5ʹ-termini. By using these primers to perform PCR amplification on the pre-synthesized first-strand cDNA, a DNA template flanked by the T7 promoter was generated. The resulting PCR product underwent TA cloning. After that, the transformed bacteria were cultivated in a liquid culture medium. Once the plasmids were extracted, the high-concentration DNA that had been purified via gel was employed as a template. In accordance with the manufacturer’s instructions, the TranscriptAid T7 High Yield Transcription Kit and the GeneJET RNA Purification Kit (both from Thermo Fisher Scientific) were used to synthesize double-stranded RNA (dsRNA). A NanoDrop 2000 spectrophotometer was utilized to measure the concentration of the dsRNA, and 1% agarose gel electrophoresis was carried out to confirm its integrity. As a control, the green fluorescent protein (GFP, U73901.1) dsRNA was synthesized in a similar fashion from the GFP plasmids stored at the Institute of Entomology, Guizhou University.

### RNAi experiment

The functional role of *SfPAGM* and *SfG6PI* were investigated via RNAi-mediated gene silencing in 1-day-old fifth-instar *S. furcifera* nymphs. Nymphs were anesthetized with CO₂ for 30 s and placed in empty Petri dishes prior to microinjection. Using a Nanoliter 2010 Injector (World Precision Instruments in Sarasota, FL, USA), around 100 ng of *SfPAGM* or *SfG6PI* dsRNA was injected into the area between the prothorax and mesothorax of each nymph. For the negative control, an equal volume of ds*GFP* was administered via injection. Each experimental treatment included three biological repeats, with 50 nymphs in each repeat group. After the injection procedure, the nymphs were raised on rice seedlings in an environment with controlled conditions: a temperature of 25 ± 2 °C, humidity of 70 ± 10%, and a light-dark cycle of 16 h light to 8 h dark. This rearing process continued until the nymphs developed into adults and emerged from their eclosion. Daily mortality was recorded for 150 nymphs allocated for phenotypic analysis. RNAi efficiency was assessed via RT-qPCR 24, 48 and 72 h post-injection using gene-specific primers (Table 1); 10 randomly selected nymphs per treatment group were analyzed. Imaging records were captured with a Keyence VH-Z20R stereomicroscope (Keyence, Osaka, Japan).

### Statistical analysis

Statistical analysis of all data was performed with SPSS 13.0 software (IBM Inc., Chicago, IL, USA). Data are expressed as the mean ± standard error (SE) from three replicates. Prior to the analysis, both normality tests and homogeneity of variance tests were carried out. The relative expression of each sample was determined using one-way analysis of variance (ANOVA) followed by Duncan’s multiple range test, with a significance level set at *P* < 0.05. For the RNAi experiments, t-tests were employed to assess significant differences in mRNA levels between each dsRNA-injected group and the dsGFP group. The effect of RNAi targeting *SfPAGM* and *SfG6PI* genes on the survival of *S. furcifera* was evaluated using Kaplan–Meier survival analysis, and the log-rank test was used to compare the survival curves of the ds*SfPAGM*, ds*SfG6PI* treatment groups and the ds*GFP* group.

## Data Availability

All data have been fully presented in the manuscript. In this study, the nucleotide and deduced amino acid sequences of the phosphoacetylglucosamine mutase (PAGM) and Glucose-6-phosphate isomerase (G6PI) genes have been deposited in the GenBank database and are accessible under the accession numbers KY381946.1 (https://www.ncbi.nlm.nih.gov/nuccore/KY381946.1/) and KY695112 ( https://www.ncbi.nlm.nih.gov/nuccore/KY695112). Further inquiries can be directed to the corresponding authors.

## References

[1] Yang, N. et al. Migration of *Sogatella furcifera* between the greater mekong subregion and northern china revealed by mtDNA and SNP. *BMC Evol. Biol.* **20**, 154 (2020). 33213363 10.1186/s12862-020-01722-4 PMC7678102

[2] Hu, S. et al. Migration sources and pathways of the pest species *Sogatella furcifera* in yunnan, china, and across the border inferred from DNA and wind analyses. *Ecol. Evol.* **10**, 8235–8250 (2020). 32788975 10.1002/ece3.6531 PMC7417236

[3] Zhou, G. H. et al. Southern rice black-streaked dwarf virus: A new proposed fijivirus species in the family reoviridae. *Chin. Sci. Bull.* **53**, 3677–3685 (2008).

[4] Matsukura, K. et al. Quantitative analysis of southern rice black-streaked dwarf virus in *Sogatella furcifera* and virus threshold for transmission. *Phytopathology* **105**, 550–554 (2015). 25870927 10.1094/PHYTO-05-14-0142-R

[5] Wu, N., Zhang, P., Liu, W., Cao, M. & Wang, X. Sequence analysis and genomic organization of a new insect iflavirus, *Sogatella furcifera* honeydew virus 1. *Arch. Virol.* **163**, 2001–2003 (2018). 29574590 10.1007/s00705-018-3817-7

[6] Yang, X. et al. Identification and RNAi-based functional analysis of four chitin deacetylase genes in *Sogatella furcifera* (Hemiptera: Delphacidae). *J. Insect Sci.* **21** (2021). 10.1093/jisesa/ieab051 PMC8325873 34333649

[7] Ren, Q. et al. Conserved micrornas mir-8-3p and mir-2a-3 targeting chitin biosynthesis to regulate the molting process of *Sogatella furcifera* (Horváth) (Hemiptera: Delphacidae). *J. Econ. Entomol.* **117**, 1675–1685 (2024). 38894631 10.1093/jee/toae123

[8] Candy, D. J. & Kilby, B. A. Studies on chitin synthesis in the desert locust. *J. Exp. Biol.* **39** (1962).

[9] Jaworski, E. W. L. M. Synthesis of chitin in cell-free extracts of *Prodenia eridania*. *Nature* **198**, 790 (1963).13978870

[10] Cohen, E. Chitin synthesis and inhibition: A revisit. *Pest Manag Sci.* **57**, 946–950 (2001). 11695188 10.1002/ps.363

[11] Merzendorfer, H. & Zimoch, L. Chitin metabolism in insects: Structure, function and regulation of chitin synthases and chitinases [review]. *J. Exp. Biol.* **206**, 4393–4412 (2003). 14610026 10.1242/jeb.00709

[12] Cai, Z., Wu, H. & Lin, T. Isolation and expression of glutamine-fructose-6-phosphate aminotransferase gene from *Monochamus alternatus*. *Southwest. China J. Agric. Sci.* **29**, 2131–2137 (2016).

[13] Kato, N., Mueller, C. R., Wessely, V., Lan, Q. & Christensen, B. M. *Aedes aegypti* phosphohexomutases and uridine diphosphate-hexose pyrophosphorylases: Comparison of primary sequences, substrate specificities and temporal transcription. *Insect Mol. Biol.* **14**, 615–624 (2005). 16313562 10.1111/j.1365-2583.2005.00592.x

[14] Liu, X. et al. Chitinase (CHI) of *Spodoptera frugiperda* affects molting development by regulating the metabolism of chitin and trehalose. *Front. Physiol.* **13**, 14 (2022). 10.3389/fphys.2022.1034926 PMC9574123 36262255

[15] Shakeel, M. et al. Knockdown of the glucosamine-6-phosphate n-acetyltransferase gene by RNA interference enhances the virulence of entomopathogenic fungi against rice leaffolder *Cnaphalocrocis medinalis*. *Pest Biochem. Physiol.* **205**, 106119 (2024). 10.1016/j.pestbp.2024.106119 39477580

[16] Zeng, B. et al. Effect of glycogen synthase and glycogen phosphorylase knockdown on the expression of glycogen- and insulin-related genes in the rice brown planthopper *Nilaparvata lugens*. *Comp. Biochem. Physiol. D*. **33**, 100652 (2020). 10.1016/j.cbd.2019.100652 31927198

[17] Palaka, B. K., Tuleshwori, D., Sapam, V. & Rao, D. *Molecular Cloning and Characterization of Phosphoacetylglucosamine Mutase from Bombyx mori* 4 (AkiNik Publications, 2017).

[18] Zhang, Z., Zhang, X., Zhang, J. & Liu, X. Expression and function of phosphoacetylglucosamine mutase gene in *Locusta migratoria*. *J. Shanxi Univ. (Nat Sci. Ed)*. **42**, 929–934 (2019).

[19] Wang, Z. et al. Molecular cloning, expression, and functional analysis of the chitin synthase 1 gene and its two alternative splicing variants in the white-backed planthopper, *Sogatella furcifera* (Hemiptera: Delphacidae). *Sci. Rep.* **1** (2019). 10.1038/s41598-018-37488-5 PMC6355952 30705372

[20] Wang, Z. et al. Molecular characterization of UDP-*N*-acetylglucosamine pyrophosphorylase and its role in the growth and development of the white-backed planthopper *Sogatella furcifera* (Hemiptera: Delphacidae). *Genes* **13**, 1340 (2022). 10.3390/genes13081340 PMC9332809 35893078

[21] Wang, Z. et al. Silencing of glutamine: Fructose-6-phosphate aminotransferase impairs growth and development in *Sogatella furcifera* (Hemiptera: Delphacidae). *Biomolecules* **13**, 1433 (2023). 10.3390/biom13101433 PMC10604220 37892115

[22] Tan, J. Y. et al. New Insights into Expanding the insecticidal spectrum of dsRNA mediated by the high sequence identity between dsRNA and nontarget mRNA. *J. Agric. Food Chem.* **73 **(8), 4605–4616 (2025). 10.1021/acs.jafc.4c12803 39948051

[23] Chen, J., Liang, Z., Liang, Y., Pang, R. & Zhang, W. Conserved micrornas mir-8-5p and mir-2a-3p modulate chitin biosynthesis in response to 20-hydroxyecdysone signaling in the brown planthopper, *Nilaparvata lugens*. *Insect Biochem. Mol. Biol.* **43**, 839–848 (2013). 23796434 10.1016/j.ibmb.2013.06.002

[24] Yang, W. et al. Two chitin biosynthesis pathway genes in *Bactrocera dorsalis* (Diptera: Tephritidae): Molecular characteristics, expression patterns, and roles in larval-pupal transition. *J. Econ. Entomol.* **108**, 2433–2442 (2015). 26453732 10.1093/jee/tov186

[25] Zhang, J. et al. Silencing of two alternative splicing-derived mRNA variants of chitin synthase 1 gene by RNAi is lethal to the oriental migratory locust, *Locusta migratoria* manilensis (Meyen). *Insect Biochem. Mol. Biol.* **40**, 824–833 (2010). 20713155 10.1016/j.ibmb.2010.08.001

[26] Li, J. et al. Functional characterization of phosphoacetylglucosamine mutase in *Bombyx mori* chitin synthesis and development. *J. Insect Physiol.* **135**, 104289 (2021).

[27] Walski, T. et al. Functional analysis of O-glycosylation-related genes in *Tribolium castaneum* development. *Front. Physiol.* **7**, 589 (2016).28018232

[28] Zhang, Y., Li, J. & Wang, X. Research progress on insect chitin metabolism pathway and its regulation mechanism. *J. Capital Norm Univ. (Nat Sci. Ed.* **45**, 1–10 (2024). (In Chinese with English abstract).

[29] Wang, Z., Zhou, C., Long, G., Yang, H. & Jin, D. Sublethal effects of buprofezin on development, reproduction, and chitin synthase 1 gene (SfCHS1) expression in the white-backed planthopper, *Sogatella furcifera* (Hemiptera: Delphacidae). *J. Asia-Pac. Entomol.* **21**, 585–591 (2018).

[30] Long, G. et al. Wing expansion functional analysis of ion transport peptide gene in *Sogatella furcifera* (Horvath) (Hemiptera: Delphacidae). *Comp. Biochem. Physiol. B*. **271**, 110946 (2024). 38266956 10.1016/j.cbpb.2024.110946

[31] Livak, K. J. & Schmittgen, T. D. Analysis of relative gene expression data using real-time quantitative PCR and the 2(-delta delta c(t)) method. *Methods* **25**, 402–408 (2001). 11846609 10.1006/meth.2001.1262

